# Supplementary material for: Cadmium exposure as a contributing factor to hepato-renal injury in apparently healthy active cigarette smokers in buea-cameroon
Source: PLoS One. 2026 Jun 9;21(6):e0333775. doi: 10.1371/journal.pone.0333775 (PMC13249404; doi:10.1371/journal.pone.0333775)
Supplement: S1 File — (DOCX) [file pone.0333775.s001.docx]

**Questionnaire**

1. **Information**

Good morning/afternoon, and thank you for taking the time to consider participating in this research study. I am a master’s student in Toxicology from the Faculty of Health Sciences, University of Buea.

We are conducting a study on the topic **ESTIMATION OF CADMIUM DAILY EXPOSURE FROM CIGARETTE AND EVALUATION OF HEPATO-RENAL INJURY IN RESPONSE TO TOBACCO CONSUMPTION AMONG APPARENTLY HEALTHY ACTIVE SMOKERS IN BUEA-CAMEROON.** The main objective of this study is to estimate exposure to cadmium from smoking, and quantify the cadmium levels present in various tobacco products, with a focus on cigarettes commonly consumed by individuals in Buea and their implication on liver and kidney function.

Your valuable responses will contribute to a deeper understanding of these issues and aid in devising strategies to mitigate the risks associated with hepato-renal injury in response to tobacco consumption.

**Participation is voluntary:** Your participation in this study is completely voluntary. You have the right to refuse to participate at any time, even if you have already begun the questionnaire.

**Confidentiality:** All information you provide will be kept strictly confidential. Your name and any other identifying information will not be shared with anyone outside of the research team, and your responses will be anonymized in any publications or reports.

**Benefits and Risks:** There are no direct benefits or risks associated with participating in this study. However, the results of your test will be returned to you.

## Consent: By completing this questionnaire, you are indicating that you have read and understood the above information and that you agree to participate in this study voluntarily.

Please ***Tick*** "Yes" below if you consent to participate. If you do not consent, please ***Tick*** "No" and the questionnaire will not continue.

Yes, I consent to participate.

No, I do not consent to participate.

Thank you for your time and consideration!

1. **DATA COLLECTION FORM.**

**Socio-demographic variables**

| **QUESTIONS** | **RESPONSES** *(****Please Tick****)* |
| --- | --- |
| Gender: | Female  Male |
| Age Range (years): | [18-25]  ]25-35]  ]35-45] ]45-55] |
| Marital status | Single  Married |
| Level of education | Primary  Secondary  Undergraduate  Postgraduate |
| Occupation | civil servant self-employed  unemployed manual labor |
|  |  |
| Level of financial income | low (< 50.000 per month)  Lower-Middle (50.000 – 100.000 per month)  middle– High (100.001 – 200.000 per month)  High (>200.000 per month) |
| Location | Rural  Urban |

**Exposure and risk factors**

| smoker | Yes  No Former |
| --- | --- |
| What brand(s) of cigarettes do you consume? |  |
| smoking history (years) | [1-5] ]5-10]  ]10-20]  >20 |
| Approximate number of cigarettes/days | [1-5] ]5-10]  ]10-20]  >20 |
| Do you smoke electronic  cigarettes? | Yes  No Former |
| How many times do you usually recharge the tank of your e-cigarette in a day? | < 2 2-5 6-9 |
| Do you use any other  tobacco products? | Yes  No Former |
|  |  |
| Please specify |  |
| Number of years used | < 2 2-5 6-9 |
| Alcohol consumption | Yes No  How often do you drink alcohol?  Daily Weekly  Monthly  Please specify the type of alcoholic beverage: wine  beer whiskey |

**Hepato-renal injury Associated pathologies** **and dietary habits**

| **Cancer** | Yes  No  If so, since when? \|_________________\| |
| --- | --- |
| **Liver damage** | Yes No  If so, since when? \|_________________\| |
| **Visual disorders** | Yes  No  If so, since when? \|_________________\| |
| **Dizziness** | Yes No  If so, since when? \|_________________\| |
| **Hypertension** | Yes No  If so, since when? _________________ |
| **Diabetes** | Yes  No  If Yes, since when? \|_________________\| |
| **Renal insufficiency** | Yes  No  If Yes, since when? \|_________________\| |
| **Nephropathy** | Yes  No  If Yes, since when? \|_________________\| |
| **Psychological disorders** | Yes  No  If yes, since when? \|_________________\| |
| **Neurological disorders** | Yes  No  If Yes, since when? \|_________________\| |
