# Supplementary material for: Cadmium exposure as a contributing factor to hepato-renal injury in apparently healthy active cigarette smokers in buea-cameroon
Source: PLoS One. 2026 Jun 9;21(6):e0333775. doi: 10.1371/journal.pone.0333775 (PMC13249404; doi:10.1371/journal.pone.0333775)
Supplement: Attachment — Submitted filename: Response to Reviewers.docx [file pone.0333775.s005.docx]

**RESPONSE TO THE REVIEWER’S COMMENTS**

**Note: All changes or suggestions are written in red in the revised manuscript**

Dear Editor-in-Chief of the journal: *PLOS ONE*

Thank you for your letter dated March 20^th^, 2026, and the opportunity given to us to revise and resubmit the manuscript entitled **“Cadmium Exposure and Hepato-renal Injury in Response to Cigarette Smoking in Apparently Healthy Active Smokers in Buea-Cameroon. PONE-D-25-50711”.** We would also like to take this opportunity to thank the editorial team and reviewers for their helpful comments, which greatly contributed to improving the current version of this manuscript. The manuscript has been updated in accordance with the reviewers' recommendations. Most of their inquiries have been answered, and some clarifications have been provided. Throughout this revised manuscript, modifications are written in red.

**Editor and Reviewer comments**

**Editor’s comment: Journal Requirements**

**Comment #1:** When submitting your revision, we need you to address these additional requirements.
1. Please ensure that your manuscript meets PLOS ONE's style requirements, including those for file naming. The PLOS ONE style templates can be found at
[https://journals.plos.org/plosone/s/file?id=wjVg/PLOSOne_formatting_sample_main_body.pdf](https://track.editorialmanager.com/CL0/https:%2F%2Fjournals.plos.org%2Fplosone%2Fs%2Ffile%3Fid=wjVg%2FPLOSOne_formatting_sample_main_body.pdf/1/010f019d08ea6048-ac472bdb-4871-47c3-8f2d-38734d68ae1d-000000/IXYng5o4s8dxmseuNfN7NNtLYe_FmyJVwkzbh5rO4jo=255) and [https://journals.plos.org/plosone/s/file?id=ba62/PLOSOne_formatting_sample_title_authors_affiliations.pdf](https://track.editorialmanager.com/CL0/https:%2F%2Fjournals.plos.org%2Fplosone%2Fs%2Ffile%3Fid=ba62%2FPLOSOne_formatting_sample_title_authors_affiliations.pdf/1/010f019d08ea6048-ac472bdb-4871-47c3-8f2d-38734d68ae1d-000000/WxCpZXRsoHV_3ja5-sWp1F8TF9RZvmKpEjWOjBg8RoQ=255)

**Author Response:** Dear Editor, thank you for your remark. The name of all files associated to this submission has been revised according to PLOS ONE’s style requirements.

**Comment #2:** 2. We note that there is identifying data in the Supporting Information file <S1_File.docx, S2_File.xlsx>. Due to the inclusion of these potentially identifying data, we have removed this file from your file inventory. Prior to sharing human research participant data, authors should consult with an ethics committee to ensure data are shared in accordance with participant consent and all applicable local laws.

Data sharing should never compromise participant privacy. It is therefore not appropriate to publicly share personally identifiable data on human research participants. The following are examples of data that should not be shared:

-Name, initials, physical address

-Ages more specific than whole numbers

-Internet protocol (IP) address

-Specific dates (birth dates, death dates, examination dates, etc.)

-Contact information such as phone number or email address

-Location data

-ID numbers that seem specific (long numbers, include initials, titled “Hospital ID”) rather than random (small numbers in numerical order)

Data that are not directly identifying may also be inappropriate to share, as in combination they can become identifying. For example, data collected from a small group of participants, vulnerable populations, or private groups should not be shared if they involve indirect identifiers (such as sex, ethnicity, location, etc.) that may risk the identification of study participants.
Additional guidance on preparing raw data for publication can be found in our Data Policy (https://journals.plos.org/plosone/s/data-availability#loc-human-research-participant-data-and-other-sensitive-data) and in the following article: http://www.bmj.com/content/340/bmj.c181.long.
Please remove or anonymize all personal information (<specific identifying information in file to be removed>), ensure that the data shared are in accordance with participant consent, and re-upload a fully anonymized data set. Please note that spreadsheet columns with personal information must be removed and not hidden as all hidden columns will appear in the published file.

**Author Response:** Dear Editor, thank you for your comments. To the best of our knowledge, the supplementary files S1 and S2 do not contain any personally identifiable data, and the participants are indicated by codes. In case, you have specifically identified one, please, kindly indicate and we will be pleased to correct.

**Comment #3:** If the reviewer comments include a recommendation to cite specific previously published works, please review and evaluate these publications to determine whether they are relevant and should be cited. There is no requirement to cite these works unless the editor has indicated otherwise

**Author Response:** Dear Editor, thank you for your suggestions. We have taken note of your suggestions.

**Reviewer’s comment**

**Reviewer #1:** Dear Editor-in-Chief, this manuscript addresses a relevant public health concern by assessing cadmium exposure from cigarette smoking and its association with liver and kidney biomarkers in Buea, Cameroon. The study design and exposure assessment are appropriate, and findings are clearly presented and biologically plausible. Only minor clarifications are needed. The manuscript is acceptable with minimal revision.

**Abstract**

**Comment #1:** I suggest authors rephrase objective to “This study, conducted in Buea (Southwest Region, Cameroon), aimed to assess cadmium inhalation exposure from cigarette smoking and its association with altered liver and kidney function biomarkers in apparently healthy smokers.”.

**Author Response:** Dear Reviewer, thank you for your suggestions. The objective has been edited in the revised manuscript as follows: “This study, conducted in Buea (Southwest Region, Cameroon), aimed to assess cadmium inhalation exposure from cigarette smoking and its association with altered liver and kidney function biomarkers in apparently healthy smokers”.

**Comment #2:** Line 34: rephrase to “A survey was used to recruit smokers and collect data on their smoking habits.”

**Author Response:** Dear Reviewer, thank you for your suggestion. It has been considered in the revised manuscript.

**Comment #3:** Line 38: replace “active” with “smokers” and Line 50: delete “active”

**Author Response:** Dear Reviewer, thank you for your remarks. In the revised manuscript, the corrections have been made as suggested.

**Introduction**

**Comment #4:** Line 54-55: rephrase to “……to many organs and systems of the body”.

**Author Response:** Dear Reviewer, thank you for your suggestion. It has been corrected.

**Comment #5:** Lines 71-72: Some vital information on tobacco consumption and health in Cameroon are missing. I suggest you cite all tobacco papers from Cameroon e.g., https://www.ijtsrd.com/papers/ijtsrd46334.pdf and <https://doi.org/10.56557/jodagh/2025/v18i29850>.

**Author Response:** Dear Reviewer, thank you for your suggestion. Additional citation has been added to update information on tobacco consumption and health in Cameroon.

**Comment #6:** Line 73: rephrase to “Cigarette smoking can harm nearly all organs and systems of the human body;” ; “Line 74: insert “which are” to read as “…smoking can damage the liver and kidneys, which are two important organs.” and Line 80: rephrase to “…can lead to liver injury and fibrosis, a condition in which liver tissue becomes thickened and scarred.”

**Author Response:** Dear Reviewer, thank you for your comment. The sentences have been rephrased.

**Comment #7:** Lines 83-85: delete “an”, and “a” to read as “...including increased blood pressure, reduced renal blood flow, glomerular damage, and a gradual loss of kidney function”.

**Author Response:** Dear Reviewer, thank you for your comment. The correction has been made in the revised manuscript.

**Comment #8:** Line 85: delete “activity” and Line 86: delete “, a state of irreversible kidney injury”.

**Author Response:** Dear Reviewer, thank you for your remarks. It has been corrected in the revised manuscript.

**Comment #9:** Line 88: replace “abnormal increased levels of blood urea and creatinine” to “abnormally elevated levels of blood urea and creatinine”.

**Author Response:** Dear Reviewer, thank you for your suggestion. It has been replaced.

**Comment #10:** Line 83: Insert the statement from Line 90-92 “In the ……[20]” in line 83 after the reference “[16]”.

**Author Response:** Dear Reviewer, thank you for your suggestion. It has been considered in the revised manuscript.

**Comment #11:** Lines 101-105: this objective is not the same as in the abstract section – make it uniform/the same.

**Author Response:** Dear Reviewer, thank you for your remark. The objective has been revised to make it uniform with the one stated in the abstract.

**Methods**

**Comment #12:** Lines 111-113: what is the association between Cd levels/exposures with seasons? Did you show that in this work? I though population size of Buea could be vital mentioning here, so readers understand the scope of the study and potential damage due to Cd from smoking – or better still insert the data on population size

**Author Response:** Thank you for this important observation. We agree that seasonal variation could influence environmental cadmium exposure, particularly through factors such as rainfall, humidity, and agricultural burning, which may affect ambient air quality and tobacco leaf cadmium content. However, in this study, we did not assess the association between cadmium exposure levels and seasons. Our investigation was a cross-sectional study conducted over a single time frame (May to October 2024), which corresponds to the long rainy season in Buea. Therefore, we were unable to account for seasonal variations throughout the year. We have now clarified this limitation in the revised manuscript.

Regarding the population size of Buea, we agree that providing this information helps readers better understand the scope and generalizability of the study. Buea is the capital of the Southwest Region of Cameroon and had an estimated population of approximately 250,000–300,000 inhabitants as of recent estimates, with a high proportion of young adults due to the presence of the University of Buea. We have now added the population data in the Study area section to contextualize the study setting and the potential public health impact of cadmium exposure from cigarette smoking in this community.

**Comment #13:** Lines 115-117: the statement “The area was therefore chosen to raise awareness among the young and old alike that smoking can potentially damage vital organs like the kidneys and the liver.” appears as if you knew the results of this work and as if the aim of your work was to raise awareness! I suggest you rephrase.

**Author Response:** Dear Reviewer, thank you for your comment. The sentence has been rephrased in the revised manuscript as follows: …The area was therefore selected due to its large young adult population, enabling us to evaluate smoking-induced liver and kidney damage, with the goal of using the findings to guide future public health awareness efforts…

**Comment #14:** Lines 118-122: some vital information/components are missing on the study design, for example, the variables (independent/dependent), sampling methods, etc. kindly consider re-writing this section.

**Author Response:** Dear Reviewer, thank you for your comments. We have rewritten the section and the missing information/components have been included in the revised manuscript. Please, refer to Page 5 and 6.

**Comment #15:** Lines 124-125: if that was the primary aim, what about the effects on hepatocellular/kidney functions etc

**Author Response:** Thank you for this important observation. You are correct that the original wording of the primary aim was incomplete, as it only mentioned estimating cadmium exposure levels without including the assessment of effects on liver and kidney functions, which is a major component of this study. We have now revised the sentence to clearly state that the study had two primary aims: (1) to estimate cadmium exposure from cigarette smoking, and (2) to assess the association between smoking and abnormal levels of liver and kidney function biomarkers (including hepatocellular injury markers such as ALT, and renal function markers such as urea, creatinine, albuminuria, and glucosuria).

**Comment #16:** Line 129: is this “Cameroon [23] (Njoumeni et al., 2020)” or “Cameroon [23]” or “Cameroon [23, 24]” reference 24 being Njoumeni et al., 2020?

**Author Response:** Dear Reviewer, thank you for your remark. The correct citation is “Cameroon [23]”. “(Njoumeni et al., 2020)” has been deleted.

**Comment #17:** Line 131: N of 78 (95) is too small for such studies! Well, lets deal with it.

**Author Response:** Dear Reviewer, thank you for this observation. We acknowledge that a sample size of 95 active smokers (derived from 78 plus 20%) may appear relatively small for epidemiological studies. However, we would like to provide the following justifications:

Prevalence-based calculation: The sample size was calculated using Cochran's formula based on the reported smoking prevalence in Cameroon (5.3%). This is a standard approach for cross-sectional studies when the population size is large and no prior data on the specific outcome are available.

Feasibility constraints in resource-limited settings: This study was conducted in Buea, Cameroon, where resources for large-scale biomonitoring studies are limited. The recruitment of 102 active smokers and 37 non-smokers (total 139 participants) was practically achievable within the study period and budget, while still allowing for meaningful statistical comparisons.

**Limitation acknowledged:** We agree that a larger sample size would enhance generalizability and allow for more robust subgroup analyses. We have now explicitly acknowledged this as a limitation in the revised manuscript (Discussion section). We have included the following sentence to the limitations paragraph: “Furthermore, although the sample size was calculated based on the local smoking prevalence and was sufficient to detect significant associations, it remains relatively modest. A larger, multi-center study would be necessary to confirm these findings and improve their generalizability to the broader Cameroonian population”.

**Comment #18:** Lines 133-137: Inclusion and exclusion criteria not well stated eg., did you include smokers with known illnesses? Line 140: Are there smoking areas in Buea??? Line 140: replace “a” with “each”

**Author Response:** Dear Reviewer, thank you for your comments. Sub-headings have been included in the revised manuscript to specifically include the “inclusion and exclusion criteria”. There are no smoking areas in Buea and the statement has been deleted. Line 140: replace “a” with “each”, corrected.

**Comment #19:** Lines 141-142: Not only is the statement too long, it has repetitions e.g. gave their consent by signing the informed consent … therefore, kindly shorten the statement and rephrase the second part of that statement from “..were clearly explained; and only participants who gave their consent by signing the informed consent were admitted to the study.” to read as “..were clearly explained. Participation in the study was limited to individuals who signed the informed consent form.”

**Author Response:** Dear Reviewer, thank you for your remark. The statement has been corrected as suggested.

**Comment #20:** Lines 138-149: What were the inclusion and exclusion criteria? This is missing from the manuscript. This information was attempted on lines 118-122.

**Author Response:** Dear Reviewer, thank you for your remark. The inclusion and exclusion criteria have been explicitly added. Please, refer to Page 6 of the revised manuscript.

**Comment #21:** Line 154: How was “pre-testing” done? I suggest you provide a statement on that. For example – “During the pre-testing phase, the questionnaire was randomly administered to five market women and ten primary school pupils in classes five and six. This process aimed to ensure that the language used in the questionnaire was simple, clear, and unambiguous, and that it could be easily understood by all participants. As more than 80% positive responses were obtained, the questionnaire was considered valid. The results of the pre-testing phase were not included in the findings of this study.”

**Author Response:** Dear Reviewer, thank you for your comment. We have now added a description of pre-testing phase according to your suggestions. Thank you very much for that useful suggestion.

**Comment #22:** Lines 151-163: The heading on line 151 does not adequately reflect the content in lines 152-163. Lines 158-163 deserve a separate subheading “Sampling of biological specimens (blood and urine)”. Meanwhile, the square brackets in Line 158 needs to be adjusted.

**Author Response:** Thank you for this careful observation. You are correct that the original heading "Identification of the commercial cigarette brands consumed in Buea" did not adequately reflect the content in lines 158–163, which describe the collection of biological specimens (blood and urine). We have now restructured this section as follows:

A new subheading "Collection of biological specimens (blood and urine)" has been introduced to cover lines 158–163; and the square bracket notation in the original line 158 has been corrected (changed from "[5-10]" to the proper range "5–10" as appropriate).

**Comment #23:** Line 164: replace “of” with “in” and Line 184: capital use “C” for the first “cadmium”

**Author Response:** Dear Reviewer, thank you for your remark. They have been corrected.

**Comment #24:** Line 186: something is wrong with the phrase “The estimation of DE (µg Cd/day) DIE (µg) and DIE (µg Cd/kg b.w. per day)” – please correct it – perhaps delete “DIE (µg)”
Line 189: this statement is a result (“Only four of the most consumed brands of cigarettes based on the responses”) and so should be taken to the results section where you would specify them. So rephrase by deleting “four of”. Line 190: put a full stop at the end of the statement.

**Author Response:** Dear Reviewer, thank you for your remarks. It has been corrected.

**Comment #25:** Lines 191-192: I think De and DIE have been defined on line 186 and need not to be defined anymore. Line 191: provide references for the formula 2 and 3 used. Line 213: provide references for the formula 5 used.

**Author Response:** Dear Reviewer, thank you for your comment. DE and DIE were defined in Line 191-192 to provide the full meaning of terms in the formula. The reference for the formula (2), (3), and (5) has been indicated as [25].

**Comment #26:** Lines 214-215: items like “DIE” which have been defined needs not to be defined again.
**Author Response:** Dear Reviewer, thank you for your remark. The items were defined to provide the full meaning of terms in the formula.

**Results
Comment #27:** Lines 262-263: age range provided in square brackets is confusing as it appears as intext citation – use normal brackets.

**Author Response:** Dear Reviewer, thank you for your comment. Please, kindly note that the square brackets here indicate age range.

**Comment #28:** Line 268 and 273: on table 1 and 2, use simple brackets and not square brackets for example - age range on table 1 and number of cigarettes for table 2 – it is confusing with intext citation.

Line 278: use simple/normal brackets and not square brackets – do same through out the manuscript to differentiate intext citations from manuscript content information

**Author Response:** Thank you for this important observation regarding the use of square brackets in Tables 1 and 2. We understand your concern that the square brackets might be confused with in-text citation formatting.

However, we would like to respectfully clarify that in this context, the square brackets are used exclusively to denote inclusive and exclusive numerical intervals (ranges), a standard mathematical notation. Specifically:

[18–25] indicates an inclusive range: all values from 18 to 25, including both endpoints (18 and 25).

]25–35] indicates a half-open interval: values greater than 25 up to and including 35.

]35–45], ]45–55], etc., follow the same logic.

This notation is commonly used in epidemiological and biostatistical reporting to precisely define age groups, smoking frequency categories, and other continuous variables without ambiguity regarding whether the boundary values are included or excluded.

**Comment #29:** Line 282: replace “1/4th” with “a quarter”. What do you mean by “nearly”? be specific! Line 294: delete the phrase “an observation was that”. Line 325: close the bracket that is opened.

**Author Response:** Dear Reviewer, thank you for your observations. The corrections have been implemented as suggested.

**Comment #30:** Lines 393-395: were these levels higher than the normal range? If not, then, although there was a difference, it doesn’t necessarily imply toxicity risk! Its good to compare with normal range first!

**Author Response:** Dear Reviewer, thank you for your observations. The levels were higher than normal ranges. the comparison has been done and the results are presented in Figure 2.

**Comment #31:** Lines 412-413: what does the figures “(37)”, “(38)”, “(39)” and “(25)” mean under the “Smoker Category” in the table headings.

**Author Response:** Dear Reviewer, thank you for your observations. The numbers in brackets indicate the number of smokers per smokers category.

**Comment #32:** Lines 457-461: On table 8 and table 9, what do you means by [0.00 - ] ??

**Author Response:** Thank you for pointing out this ambiguity in Tables 8 and 9.

You are correct that the notation [0.00 – ] requires clarification. Here is our explanation:

In Tables 8 and 9, the crude odds ratio (cOR) for certain categorical variables (e.g., gender: female vs. male; age range: [18–25] vs. >35) was calculated as **0.00**. This occurred because the contingency table contained a zero cell — specifically, no individuals in the "High" category for the reference group (e.g., zero females with high urea levels in Table 8, or zero participants in the >35 age group with high creatinine levels in Table 9).

When a zero cell is present, the cOR becomes zero, and the upper limit of the 95% confidence interval cannot be reliably estimated by the logistic regression model. In such cases, statistical software (SPSS) outputs a missing or infinite value for the upper bound, which we have represented as a blank or dash [0.00 – ] in the table.

To improve clarity for readers, we propose the following footnote to tables 8 and 9 explaining this notation in the revised manuscript.

"[0.00 – ] indicates that the crude odds ratio (cOR) is 0.00, and the upper limit of the 95% confidence interval could not be estimated due to the presence of a zero cell in the contingency table."

**Discussion**

**Comment #33:** Lines 472-473: replace “these young individuals” with “smokers”
Overall, proofread the entire manuscript for grammer

**Author Response:** Dear Reviewer, thank you for your observations. The corrections have been implemented as suggested.

**Reviewer #2:** In addition to Cadmium (Cd), cigarette smoke also contains PAHs: Polycyclic Aromatic e.g. B(a)P: Benzo (a) pyrene, B(a)A: Benzo (a) anthracene and TSNAs: Tobacco Specific Nitrosamines e.g. NNN: Nitrosonornicotine, NNK:4-(methylnitrosamine)-1-(3-pyridyl)-1-butanone etc and Volatile components: CO, hydrocyanide, methane, ethylene, phenol, 1,3-butadiene etc. Authors must acknowledge presence of PAHs and aromatic amines as co-toxicants and confounders and state that combined exposure is likely to potentiates organ dysfunction.

**Comment #1:** Authors have observed alterations in serum ALT, urea, creatinine, albuminuria and glucosuria level which may be reflected with PAHs and aromatic amines toxicity. To distinguish cigarette smoke inhalation associated cadmium (Cd)-specific toxicity from effects of PAHs and aromatic amines also present in cigarette smoke, authors must rely on a combination of biomonitoring, mechanistic biomarkers, experimental modeling, and statistical adjustment. For this they must perform more experiments and add results.

Below is a structured scientific approach suitable to incorporate some of the following experiments and results in the manuscript.

1. DNA adduct formation, carcinogenesis mutagenesis via CYP 450 activation Hence, it may be checked as cadmium does not form DNA adducts like PAHs and aromatic amines

2. Measuring blood cadmium level (provide recent exposure) and urinary cadmium (will provide body burden). Some of the other cadmium specific biomarkers are metallothionein levels, renal tubular dysfunction, urinary β2-microglobulin, N-acetyl-β-D-glucosaminidase (NAG), Kidney Injury Molecule-1 (KIM-1) etc. Results of some of above major tests may be incorporated in the manuscript.

**Author Response:** Dear reviewer, thank you for this insightful and scientifically rigorous comment. We fully agree that distinguishing cadmium-specific toxicity from the effects of other cigarette smoke constituents (such as PAHs and aromatic amines) would require additional biomonitoring and mechanistic biomarkers, including blood/urinary cadmium levels, metallothionein, β2-microglobulin, NAG, KIM-1, and DNA adduct analysis.

However, due to logistical and financial constraints in our resource-limited setting (Cameroon), we were unable to perform these additional experiments at the time of this study. Nevertheless, we acknowledge this as a significant limitation. To address the concern, we have added the following sentences to the Discussion section of the revised manuscript:

*"We also recognize that cigarette smoke contains numerous toxicants beyond cadmium, including polycyclic aromatic hydrocarbons (PAHs) and aromatic amines, which may also contribute to the observed hepato-renal alterations. Distinguishing cadmium-specific effects would require additional biomonitoring (e.g., blood and urinary cadmium levels), mechanistic biomarkers (e.g., metallothionein, β2-microglobulin), and DNA adduct analysis [4,5]. Additional bioassays incorporating these measurements are needed to identify the specific contribution of cadmium to smoking-induced liver and kidney injury."*

We thank you for this valuable recommendation, which will guide our future research.

**Comment #2:** Brand names of cigarette must bot be written, instead they can be coded like A, B, C,

**Author Response:** Dear reviewer, thank you for this suggestion. However, we respectfully prefer to retain the original brand names for the following reasons:

1. Public health transparency: Identifying specific cigarette brands by name informs consumers and public health authorities about which products contribute to higher cadmium exposure. This aligns with the journal's commitment to research that can directly benefit public health policy and awareness.
2. Reproducibility and contextual relevance: Naming the brands allows other researchers in Cameroon and similar settings to replicate our study or compare findings using the same products. Coding brands as A, B, C would obscure this important contextual information.
3. No commercial endorsement: We are not promoting any brand; rather, we are reporting objective analytical data on cadmium content. Our findings are critical for risk assessment and harm reduction messaging.
4. Precedent in literature: Leading journals routinely publish cigarette brand names in studies of toxic metal content (e.g., Benson et al., 2017; Caruso et al., 2013).

Caruso R, O’Connor R, Stephens W, Cummings K, Fong G. Toxic Metal Concentrations in Cigarettes Obtained from U.S. Smokers in 2009: Results from the International Tobacco Control (ITC) United States Survey Cohort. Int J Environ Res Public Health. 2013;11: 202–217. doi:10.3390/ijerph110100202.

Benson NU, Anake WU, Adedapo AE, Fred-Ahmadu OH, Ayejuyo OO. Toxic metals in cigarettes and human health risk assessment associated with inhalation exposure. Environ Monit Assess. 2017;189: 619. doi:10.1007/s10661-017-6348-x

We believe our study should follow this standard.

We have added a clarifying statement in the manuscript: *"Brand names are reported solely for scientific transparency and public health awareness, without any commercial endorsement."*

Thank you for your understanding.

The authors are grateful to the reviewers for their valuable contributions which significantly improved the quality of this work. We very much hope the revised manuscript is accepted for publication in your Journal. Thank you for your consideration.

Sincerely yours,

Corresponding author

Kouam Fondjo Arnaud
