# Supplementary material for: Cadmium exposure as a contributing factor to hepato-renal injury in apparently healthy active cigarette smokers in buea-cameroon
Source: PLoS One. 2026 Jun 9;21(6):e0333775. doi: 10.1371/journal.pone.0333775 (PMC13249404; doi:10.1371/journal.pone.0333775)
Supplement: Attachment — Submitted filename: Lakshmi Bala_comments.docx [file pone.0333775.s004.docx]

**Letter to the Editor**

The current title is understandable, but it has a conceptual issue: it singles out **cadmium exposure** while the exposure source (cigarette smoke) is actually a **mixture of multiple toxicants** (e.g., PAHs, aromatic amines, heavy metals). This creates a mismatch between **specificity (cadmium)** and **actual exposure complexity (cigarette smoke)**.

So, as it stands, the title may be **scientifically misleading or too narrow** unless the study specifically isolates cadmium’s role from other co-toxicants (e.g., through various other parameters pointed out earlier).

The key concern to me is authors have not separately analysed PAHs and aromatic amines, so they should avoid implying cadmium as the sole or primary causative factor.

Better options could be to keep cadmium but clarify context by modifying title to

**“Cadmium Exposure as a Contributor to Hepatorenal Injury in Apparently Healthy Active Smokers in Buea, Cameroon”**

**Letter to the authors**

In context of the presence of co-toxicants in addition to Cadmium (Cd) in cigarette smoke as PAHs

(Polycyclic Aromatic Hydrocarbons) e.g. B(a)P: Benzo (a) pyrene, B(a)A: Benzo (a) anthracene and

TSNAs (Tobacco Specific Nitrosamines) e.g. NNN: Nitrosonornicotine, NNK:4(methylnitrosamine)-1-(3-pyridyl)-1-butanone etc and Volatile components as CO, hydrocyanide, methane, ethylene, phenol, 1,3-butadiene etc. The cadmium exposure stands as one of the contributing factor for the observed hepato-renal injury in apparently healthy individuals.

1. It is recommended that the “Title” should be changed as:

**“Cadmium exposure as a contributing factor causing hepato-renal Injury in apparently healthy active cigarette smokers in Buea-Cameroon.”**

2. Authors must acknowledge and explicitly write and incorporate it at various places (e.g. in Abstract, Introduction, Methods and Discussion) in addition to cadmium, presence of PAHs and aromatic amines etc. (as mentioned above) as co-toxicants and confounders. It should be stated that combined exposure may likely to potentiates/contribute organ dysfunction. However, in a resource-limited setting (Cameroon), this study acknowledges a significant limitation to evaluate the effect of other co-toxicants on liver and kidney. The present revolves around evaluation of cadmium exposure of individuals while active cigarette smoking and its effect on hepatic and renal functions.

3. In the conclusion, it should be added that to distinguish cadmium-specific effects from effects caused by PAHs and aromatic amines would require additional biomonitoring (e.g., blood and urinary cadmium levels), mechanistic biomarkers (e.g., metallothionein, β_2_microglobulin), and DNA adduct analysis [4,5]. Additional bioassays incorporating these measurements are needed to identify the specific contribution of cadmium to smoking-induced liver and kidney injury. Exposure to cadmium may cause carcinogenesis/mutagenesis via CYP 450 activation and may not directly form stable DNA adduct but may induce oxidative stress and interfere with DNA repair mechanisms.
