# Supplementary material for: Cadmium exposure as a contributing factor to hepato-renal injury in apparently healthy active cigarette smokers in buea-cameroon
Source: PLoS One. 2026 Jun 9;21(6):e0333775. doi: 10.1371/journal.pone.0333775 (PMC13249404; doi:10.1371/journal.pone.0333775)
Supplement: Attachment — Submitted filename: Response_to_Reviewers_auresp_2.docx [file pone.0333775.s006.docx]

**RESPONSE TO THE REVIEWER’S COMMENTS**

**Note: All NEW changes or suggestions are written in Green in the revised manuscript**

Dear Editor-in-Chief of the journal: *PLOS ONE*

Thank you for your letter dated May 08^th^, 2026, and the opportunity given to us to revise and resubmit the manuscript entitled **“Cadmium Exposure and Hepato-renal Injury in Response to Cigarette Smoking in Apparently Healthy Active Smokers in Buea-Cameroon. PONE-D-25-50711”.** We would also like to take this opportunity to thank the editorial team and reviewers for their helpful comments, which greatly contributed to improving the current version of this manuscript. The manuscript has been updated in accordance with the reviewers' recommendations. Most of their inquiries have been answered, and some clarifications have been provided. Throughout this revised version of the manuscript, new modifications are written in green.

**Reviewer’s comment**

**Reviewer #2:** In context of the presence of co-toxicants in addition to Cadmium (Cd) in cigarette smoke as PAHs (Polycyclic Aromatic Hydrocarbons) e.g. B(a)P: Benzo (a) pyrene, B(a)A: Benzo (a) anthracene and TSNAs (Tobacco Specific Nitrosamines) e.g. NNN: Nitrosonornicotine, NNK:4(methylnitrosamine)-1-(3-pyridyl)-1-butanone etc and Volatile components as CO, hydrocyanide, methane, ethylene, phenol, 1,3-butadiene etc. The cadmium exposure stands as one of the contributing factor for the observed hepato-renal injury in apparently healthy individuals.

**Comment #1:** It is recommended that the “Title” should be changed as:

**“Cadmium exposure as a contributing factor causing hepato-renal Injury in apparently healthy active cigarette smokers in Buea-Cameroon.”**

**Author Response:** We thank the reviewer for this important suggestion. We agree that the revised title more accurately reflects that cadmium is one of several contributing factors to hepato-renal injury, rather than the sole cause. We have therefore changed the title as recommended.

**Comment #2:** Authors must acknowledge and explicitly write and incorporate it at various places (e.g. in Abstract, Introduction, Methods and Discussion) in addition to cadmium, presence of PAHs and aromatic amines etc. (as mentioned above) as co-toxicants and confounders. It should be stated that combined exposure may likely to potentiate/contribute organ dysfunction. However, in a resource-limited setting (Cameroon), this study acknowledges a significant limitation to evaluate the effect of other co-toxicants on liver and kidney. The present revolves around evaluation of cadmium exposure of individuals while active cigarette smoking and its effect on hepatic and renal functions.

**Author Response:** We have carefully incorporated this acknowledgment at multiple sections of the manuscript as requested. Specific additions have been made to the Abstract, Introduction, Methods, and Discussion sections. Throughout the revised manuscript, modifications are written in green.

**Comment #3:** In the conclusion, it should be added that to distinguish cadmium-specific effects from effects caused by PAHs and aromatic amines would require additional biomonitoring (e.g., blood and urinary cadmium levels), mechanistic biomarkers (e.g., metallothionein, β_2_microglobulin), and DNA adduct analysis [4,5]. Additional bioassays incorporating these measurements are needed to identify the specific contribution of cadmium to smoking-induced liver and kidney injury. Exposure to cadmium may cause carcinogenesis/mutagenesis via CYP 450 activation and may not directly form stable DNA adduct but may induce oxidative stress and interfere with DNA repair mechanisms.

**Author Response:** Dear Reviewer, thank you for your suggestions. We have added the recommended text to the Conclusion section.

The authors are grateful to the reviewers for their valuable contributions which significantly improved the quality of this work. We very much hope the revised manuscript is accepted for publication in your Journal. Thank you for your consideration.

Sincerely yours,

Corresponding author

Kouam Fondjo Arnaud
