# Supplementary material for: Cadmium exposure as a contributing factor to hepato-renal injury in apparently healthy active cigarette smokers in buea-cameroon
Source: PLoS One. 2026 Jun 9;21(6):e0333775. doi: 10.1371/journal.pone.0333775 (PMC13249404; doi:10.1371/journal.pone.0333775)
Supplement: Peer review history

# Peer review: Decision Letter 0

**DOI:** 10.1371/journal.pone.0333775.r001

**Authors:** Iman Al-Saleh

19 Mar 2026

PONE-D-25-50711

Cadmium Exposure and Hepato-renal Injury in Response to Cigarette Smoking in Apparently Healthy Active Smokers in Buea-Cameroon

PLOS One

Dear Dr. Kouam,

Thank you for submitting your manuscript to PLOS ONE. Two expert reviewers in the field have evaluated the manuscript and provided constructive feedback that will enhance its quality. Therefore, we invite you to submit a revised version of the manuscript that addresses the points raised during the review process.

Please submit your revised manuscript by May 03 2026 11:59PM. If you will need more time than this to complete your revisions, please reply to this message or contact the journal office at plosone@plos.org. When you're ready to submit your revision, log on to https://www.editorialmanager.com/pone/ and select the 'Submissions Needing Revision' folder to locate your manuscript file.

Please include the following items when submitting your revised manuscript:

- A letter that responds to each point raised by the academic editor and reviewer(s). You should upload this letter as a separate file labeled 'Response to Reviewers'.
- A marked-up copy of your manuscript that highlights changes made to the original version. You should upload this as a separate file labeled 'Revised Manuscript with Track Changes'.
- An unmarked version of your revised paper without tracked changes. You should upload this as a separate file labeled 'Manuscript'.

If you would like to make changes to your financial disclosure, please include your updated statement in your cover letter. Guidelines for resubmitting your figure files are available below the reviewer comments at the end of this letter.

If applicable, we recommend that you deposit your laboratory protocols in protocols.io to enhance the reproducibility of your results. Protocols.io assigns your protocol its own identifier (DOI) so that it can be cited independently in the future. For instructions see: https://journals.plos.org/plosone/s/submission-guidelines#loc-laboratory-protocols. Additionally, PLOS ONE offers an option for publishing peer-reviewed Lab Protocol articles, which describe protocols hosted on protocols.io. Read more information on sharing protocols at https://plos.org/protocols?utm_medium=editorial-email&utm_source=authorletters&utm_campaign=protocols.

We look forward to receiving your revised manuscript.

Kind regards,

Iman Al-Saleh

Academic Editor

PLOS One

Journal Requirements:

When submitting your revision, we need you to address these additional requirements.

1. Please ensure that your manuscript meets PLOS ONE's style requirements, including those for file naming. The PLOS ONE style templates can be found at

https://journals.plos.org/plosone/s/file?id=wjVg/PLOSOne_formatting_sample_main_body.pdf and https://journals.plos.org/plosone/s/file?id=ba62/PLOSOne_formatting_sample_title_authors_affiliations.pdf

2. We note that there is identifying data in the Supporting Information file <S1_File.docx, S2_File.xlsx>. Due to the inclusion of these potentially identifying data, we have removed this file from your file inventory. Prior to sharing human research participant data, authors should consult with an ethics committee to ensure data are shared in accordance with participant consent and all applicable local laws.

Data sharing should never compromise participant privacy. It is therefore not appropriate to publicly share personally identifiable data on human research participants. The following are examples of data that should not be shared:

-Name, initials, physical address

-Ages more specific than whole numbers

-Internet protocol (IP) address

-Specific dates (birth dates, death dates, examination dates, etc.)

-Contact information such as phone number or email address

-Location data

-ID numbers that seem specific (long numbers, include initials, titled “Hospital ID”) rather than random (small numbers in numerical order)

Data that are not directly identifying may also be inappropriate to share, as in combination they can become identifying. For example, data collected from a small group of participants, vulnerable populations, or private groups should not be shared if they involve indirect identifiers (such as sex, ethnicity, location, etc.) that may risk the identification of study participants.

Additional guidance on preparing raw data for publication can be found in our Data Policy (https://journals.plos.org/plosone/s/data-availability#loc-human-research-participant-data-and-other-sensitive-data) and in the following article: http://www.bmj.com/content/340/bmj.c181.long.

Please remove or anonymize all personal information (<specific identifying information in file to be removed>), ensure that the data shared are in accordance with participant consent, and re-upload a fully anonymized data set. Please note that spreadsheet columns with personal information must be removed and not hidden as all hidden columns will appear in the published file.

3. If the reviewer comments include a recommendation to cite specific previously published works, please review and evaluate these publications to determine whether they are relevant and should be cited. There is no requirement to cite these works unless the editor has indicated otherwise.

[Note: HTML markup is below. Please do not edit.]

Reviewers' comments:

Reviewer's Responses to Questions

**Comments to the Author**

1. Is the manuscript technically sound, and do the data support the conclusions?

The manuscript must describe a technically sound piece of scientific research with data that supports the conclusions. Experiments must have been conducted rigorously, with appropriate controls, replication, and sample sizes. The conclusions must be drawn appropriately based on the data presented.

Reviewer #1: Yes

Reviewer #2: Partly

**********

2. Has the statistical analysis been performed appropriately and rigorously?

Reviewer #1: Yes

Reviewer #2: Yes

**********

3. Have the authors made all data underlying the findings in their manuscript fully available?

The PLOS Data policy requires authors to make all data underlying the findings described in their manuscript fully available without restriction, with rare exception (please refer to the Data Availability Statement in the manuscript PDF file). The data should be provided as part of the manuscript or its supporting information, or deposited to a public repository. For example, in addition to summary statistics, the data points behind means, medians and variance measures should be available. If there are restrictions on publicly sharing data—e.g. participant privacy or use of data from a third party—those must be specified.

Reviewer #1: Yes

Reviewer #2: Yes

**********

4. Is the manuscript presented in an intelligible fashion and written in standard English?

PLOS ONE does not copyedit accepted manuscripts, so the language in submitted articles must be clear, correct, and unambiguous. Any typographical or grammatical errors should be corrected at revision, so please note any specific errors here.

Reviewer #1: Yes

Reviewer #2: Yes

**********

5. Review Comments to the Author

Please use the space provided to explain your answers to the questions above. You may also include additional comments for the author, including concerns about dual publication, research ethics, or publication ethics. (Please upload your review as an attachment if it exceeds 20,000 characters)

Reviewer #1: Reviewer’s Comments

Cadmium Exposure and Hepato-renal Injury in Response to Cigarette Smoking in Apparently Healthy Active Smokers in Buea-Cameroon.

PONE-D-25-50711

Dear Editor-in-Chief,

This manuscript addresses a relevant public health concern by assessing cadmium exposure from cigarette smoking and its association with liver and kidney biomarkers in Buea, Cameroon. The study design and exposure assessment are appropriate, and findings are clearly presented and biologically plausible. Only minor clarifications are needed. The manuscript is acceptable with minimal revision.

Comments:

Abstract

Lines 31-34: I suggest authors rephrase objective to “This study, conducted in Buea (Southwest Region, Cameroon), aimed to assess cadmium inhalation exposure from cigarette smoking and its association with altered liver and kidney function biomarkers in apparently healthy smokers.”.

Line 34: rephrase to “A survey was used to recruit smokers and collect data on their smoking habits.”

Lines 34-35: rephrase to “Cadmium contents in the major cigarette brands selected were used to estimate daily cadmium exposure (DE)…..”

Line 38: replace “active” with “smokers”

Line 50: delete “active”

Introduction

Line 54-55: rephrase to “……to many organs and systems of the body” [1].

Lines 71-72: Some vital information on tobacco consumption and health in Cameroon are missing. I suggest you cite all tobacco papers from Cameroon e.g., https://www.ijtsrd.com/papers/ijtsrd46334.pdf and https://doi.org/10.56557/jodagh/2025/v18i29850

Line 73: rephrase to “Cigarette smoking can harm nearly all organs and systems of the human body.”

Line 74: insert “which are” to read as “…smoking can damage the liver and kidneys, which are two important organs..”

Line 80: rephrase to “…can lead to liver injury and fibrosis, a condition in which liver tissue becomes thickened and scarred.”

Lines 83-85: delete “an”, and “a” to read as “...including increased blood pressure, reduced renal blood flow, glomerular damage, and a gradual loss of kidney function”

Line 85: delete “activity”

Line 86: delete “, a state of irreversible kidney injury”

Line 88: replace “abnormal increased levels of blood urea and creatinine” to “abnormally elevated levels of blood urea and creatinine”

Line 83: Insert the statement from Line 90-92 “In the ……[20]” in line 83 after the reference “[16]”.

Lines 101-105: this objective is not the same as in the abstract section – make it uniform/the same.

Methods

Lines 111-113: what is the association between Cd levels/exposures with seasons? Did you show that in this work? I though population size of Buea could be vital mentioning here, so readers understand the scope of the study and potential damage due to Cd from smoking – or better still insert the data on population size

Lines 115-117: the statement “The area was therefore 116 chosen to raise awareness among the young and old alike that smoking can potentially damage vital 117 organs like the kidneys and the liver.” appears as if you knew the results of this work and as if the aim of your work was to raise awareness! I suggest you rephrase.

Lines 118-122: some vital information/components are missing on the study design, for example, the variables (independent/dependent), sampling methods, etc. kindly consider re-writing this section.

Lines 124-125: if that was the primary aim, what about the effects on hepatocellular/kidney functions etc

Line 129: is this “Cameroon [23] (Njoumeni et al., 2020)” or “Cameroon [23]” or “Cameroon [23, 24]” reference 24 being Njoumeni et al., 2020?

Line 131: N of 78 (95) is too small for such studies! Well, lets deal with it.

Lines 133-137: Inclusion and exclusion criteria not well stated eg., did you include smokers with known illnesses?

Line 140: Are there smoking areas in Buea???

Line 140: replace “a” with “each”

Lines 141-142: Not only is the statement too long, it has repetitions e.g. gave their consent by signing the informed consent … therefore, kindly shorten the statement and rephrase the second part of that statement from “..were clearly explained; and only participants who gave their consent by signing the informed consent were admitted to the study.” to read as “..were clearly explained. Participation in the study was limited to individuals who signed the informed consent form.”

Lines 138-149: What were the inclusion and exclusion criteria? This is missing from the manuscript. This information was attempted on lines 118-122.

Line 154: How was “pre-testing” done? I suggest you provide a statement on that. For example – “During the pre-testing phase, the questionnaire was randomly administered to five market women and ten primary school pupils in classes five and six. This process aimed to ensure that the language used in the questionnaire was simple, clear, and unambiguous, and that it could be easily understood by all participants. As more than 80% positive responses were obtained, the questionnaire was considered valid. The results of the pre-testing phase were not included in the findings of this study.”

Lines 151-163: The heading on line 151 does not adequately reflect the content in lines 152-163. Lines 158-163 deserve a separate subheading “Sampling of biological specimens (blood and urine)”. Meanwhile, the square brackets in Line 158 needs to be adjusted.

Line 164: replace “of” with “in”

Line 184: capital use “C” for the first “cadmium”

Line 186: something is wrong with the phrase “The estimation of DE (µg Cd/day) DIE (µg) and DIE (µg Cd/kg b.w. per day)” – please correct it – perhaps delete “DIE (µg)”

Line 189: this statement is a result (“Only four of the most consumed brands of cigarettes based on the responses”) and so should be taken to the results section where you would specify them. So rephrase by deleting “four of”

Line 190: put a full stop at the end of the statement.

Lines 191-192: I think De and DIE have been defined on line 186 and need not to be defined anymore.

Line 191: provide references for the formula 2 and 3 used.

Line 213: provide references for the formula 5 used.

Lines 214-215: items like “DIE” which have been defined needs not to be defined again.

Results

Lines 262-163: age range provided in square brackets is confusing as it appears as intext citation – use normal brackets.

Line 268 and 273: on table 1 and 2, use simple brackets and not square brackets for example - age range on table 1 and number of cigarettes for table 2 – it is confusing with intext citation

Line 278: use simple/normal brackets and not square brackets – do same through out the manuscript to differentiate intext citations from manuscript content information

Line 282: replace “1/4th” with “a quarter”. What do you mean by “nearly”? be specific!

Line 294: delete the phrase “an observation was that”

Line 325: close the bracket that is opened.

Lines 393-395: were these levels higher than the normal range? If not, then, although there was a difference, it doesn’t necessarily imply toxicity risk! Its good to compare with normal range first!

Lines 412-413: what does the figures “(37)”, “(38)”, “(39)” and “(25)” mean under the “Smoker Category” in the table headings.

Lines 457-461: On table 8 and table 9, what do you means by [0.00 - ] ??

Discussion

Lines 472-473: replace “these young individuals” with “smokers”

Overall, proofread the entire manuscript for grammer

Reviewer #2: In addition to Cadmium (Cd), cigarette smoke also contains PAHs: Polycyclic Aromatic e.g. B(a)P: Benzo (a) pyrene, B(a)A: Benzo (a) anthracene and TSNAs: Tobacco Specific Nitrosamines e.g. NNN: Nitrosonornicotine, NNK:4-(methylnitrosamine)-1-(3-pyridyl)-1-butanone etc and Volatile components: CO, hydrocyanide, methane, ethylene, phenol, 1,3-butadiene etc. Authors must acknowledge presence of PAHs and aromatic amines as co-toxicants and confounders and state that combined exposure is likely to potentiates organ dysfunction.

Authors have observed alterations in serum ALT, urea, creatinine, albuminuria and glucosuria level which may be reflected with PAHs and aromatic amines toxicity. To distinguish cigarette smoke inhalation associated cadmium (Cd)-specific toxicity from effects of PAHs and aromatic amines also present in cigarette smoke, authors must rely on a combination of biomonitoring, mechanistic biomarkers, experimental modeling, and statistical adjustment. For this they must perform more experiments and add results.

Below is a structured scientific approach suitable to incorporate some of the following experiments and results in the manuscript.

1. DNA adduct formation, carcinogenesis mutagenesis via CYP 450 activation Hence, it may be checked as cadmium does not form DNA adducts like PAHs and aromatic amines

2. Measuring blood cadmium level (provide recent exposure) and urinary cadmium (will provide body burden). Some of the other cadmium specific biomarkers are metallothionein levels, renal tubular dysfunction, urinary β2-microglobulin, N-acetyl-β-D-glucosaminidase (NAG), Kidney Injury Molecule-1 (KIM-1) etc.

Results of some of above major tests may be incorporated in the manuscript.

Brand names of cigarette must bot be written, instead they can be coded like A, B, C .....

**********

6. PLOS authors have the option to publish the peer review history of their article (what does this mean?). If published, this will include your full peer review and any attached files.

If you choose “no”, your identity will remain anonymous but your review may still be made public.

**Do you want your identity to be public for this peer review?** For information about this choice, including consent withdrawal, please see our Privacy Policy.

Reviewer #1: **Yes:** Wilfred Angie Abia

Reviewer #2: **Yes:** Prof.(Dr.) Lakshmi Bala

**********

[NOTE: If reviewer comments were submitted as an attachment file, they will be attached to this email and accessible via the submission site. Please log into your account, locate the manuscript record, and check for the action link "View Attachments". If this link does not appear, there are no attachment files.]

To ensure your figures meet our technical requirements, please review our figure guidelines: https://journals.plos.org/plosone/s/figures

You may also use PLOS’s free figure tool, NAAS, to help you prepare publication quality figures: https://journals.plos.org/plosone/s/figures#loc-tools-for-figure-preparation.

NAAS will assess whether your figures meet our technical requirements by comparing each figure against our figure specifications.

# Author comment: Author response to Decision Letter 1

**DOI:** 10.1371/journal.pone.0333775.r002

25 Apr 2026

RESPONSE TO THE REVIEWER’S COMMENTS

Note: All changes or suggestions are written in red in the revised manuscript

Dear Editor-in-Chief of the journal: PLOS ONE

Thank you for your letter dated March 20th, 2026, and the opportunity given to us to revise and resubmit the manuscript entitled “Cadmium Exposure and Hepato-renal Injury in Response to Cigarette Smoking in Apparently Healthy Active Smokers in Buea-Cameroon. PONE-D-25-50711”. We would also like to take this opportunity to thank the editorial team and reviewers for their helpful comments, which greatly contributed to improving the current version of this manuscript. The manuscript has been updated in accordance with the reviewers' recommendations. Most of their inquiries have been answered, and some clarifications have been provided. Throughout this revised manuscript, modifications are written in red.

Editor and Reviewer comments

Editor’s comment: Journal Requirements

Comment #1: When submitting your revision, we need you to address these additional requirements.

1. Please ensure that your manuscript meets PLOS ONE's style requirements, including those for file naming. The PLOS ONE style templates can be found at

https://journals.plos.org/plosone/s/file?id=wjVg/PLOSOne_formatting_sample_main_body.pdf and https://journals.plos.org/plosone/s/file?id=ba62/PLOSOne_formatting_sample_title_authors_affiliations.pdf

Author Response: Dear Editor, thank you for your remark. The name of all files associated to this submission has been revised according to PLOS ONE’s style requirements.

Comment #2: 2. We note that there is identifying data in the Supporting Information file <S1_File.docx, S2_File.xlsx>. Due to the inclusion of these potentially identifying data, we have removed this file from your file inventory. Prior to sharing human research participant data, authors should consult with an ethics committee to ensure data are shared in accordance with participant consent and all applicable local laws.

Data sharing should never compromise participant privacy. It is therefore not appropriate to publicly share personally identifiable data on human research participants. The following are examples of data that should not be shared:

-Name, initials, physical address

-Ages more specific than whole numbers

-Internet protocol (IP) address

-Specific dates (birth dates, death dates, examination dates, etc.)

-Contact information such as phone number or email address

-Location data

-ID numbers that seem specific (long numbers, include initials, titled “Hospital ID”) rather than random (small numbers in numerical order)

Data that are not directly identifying may also be inappropriate to share, as in combination they can become identifying. For example, data collected from a small group of participants, vulnerable populations, or private groups should not be shared if they involve indirect identifiers (such as sex, ethnicity, location, etc.) that may risk the identification of study participants.

Additional guidance on preparing raw data for publication can be found in our Data Policy (https://journals.plos.org/plosone/s/data-availability#loc-human-research-participant-data-and-other-sensitive-data) and in the following article: http://www.bmj.com/content/340/bmj.c181.long.

Please remove or anonymize all personal information (<specific identifying information in file to be removed>), ensure that the data shared are in accordance with participant consent, and re-upload a fully anonymized data set. Please note that spreadsheet columns with personal information must be removed and not hidden as all hidden columns will appear in the published file.

Author Response: Dear Editor, thank you for your comments. To the best of our knowledge, the supplementary files S1 and S2 do not contain any personally identifiable data, and the participants are indicated by codes. In case, you have specifically identified one, please, kindly indicate and we will be pleased to correct.

Comment #3: If the reviewer comments include a recommendation to cite specific previously published works, please review and evaluate these publications to determine whether they are relevant and should be cited. There is no requirement to cite these works unless the editor has indicated otherwise

Author Response: Dear Editor, thank you for your suggestions. We have taken note of your suggestions.

Reviewer’s comment

Reviewer #1: Dear Editor-in-Chief, this manuscript addresses a relevant public health concern by assessing cadmium exposure from cigarette smoking and its association with liver and kidney biomarkers in Buea, Cameroon. The study design and exposure assessment are appropriate, and findings are clearly presented and biologically plausible. Only minor clarifications are needed. The manuscript is acceptable with minimal revision.

Abstract

Comment #1: I suggest authors rephrase objective to “This study, conducted in Buea (Southwest Region, Cameroon), aimed to assess cadmium inhalation exposure from cigarette smoking and its association with altered liver and kidney function biomarkers in apparently healthy smokers.”.

Author Response: Dear Reviewer, thank you for your suggestions. The objective has been edited in the revised manuscript as follows: “This study, conducted in Buea (Southwest Region, Cameroon), aimed to assess cadmium inhalation exposure from cigarette smoking and its association with altered liver and kidney function biomarkers in apparently healthy smokers”.

Comment #2: Line 34: rephrase to “A survey was used to recruit smokers and collect data on their smoking habits.”

Author Response: Dear Reviewer, thank you for your suggestion. It has been considered in the revised manuscript.

Comment #3: Line 38: replace “active” with “smokers” and Line 50: delete “active”

Author Response: Dear Reviewer, thank you for your remarks. In the revised manuscript, the corrections have been made as suggested.

Introduction

Comment #4: Line 54-55: rephrase to “……to many organs and systems of the body”.

Author Response: Dear Reviewer, thank you for your suggestion. It has been corrected.

Comment #5: Lines 71-72: Some vital information on tobacco consumption and health in Cameroon are missing. I suggest you cite all tobacco papers from Cameroon e.g., https://www.ijtsrd.com/papers/ijtsrd46334.pdf and https://doi.org/10.56557/jodagh/2025/v18i29850.

Author Response: Dear Reviewer, thank you for your suggestion. Additional citation has been added to update information on tobacco consumption and health in Cameroon.

Comment #6: Line 73: rephrase to “Cigarette smoking can harm nearly all organs and systems of the human body;” ; “Line 74: insert “which are” to read as “…smoking can damage the liver and kidneys, which are two important organs.” and Line 80: rephrase to “…can lead to liver injury and fibrosis, a condition in which liver tissue becomes thickened and scarred.”

Author Response: Dear Reviewer, thank you for your comment. The sentences have been rephrased.

Comment #7: Lines 83-85: delete “an”, and “a” to read as “...including increased blood pressure, reduced renal blood flow, glomerular damage, and a gradual loss of kidney function”.

Author Response: Dear Reviewer, thank you for your comment. The correction has been made in the revised manuscript.

Comment #8: Line 85: delete “activity” and Line 86: delete “, a state of irreversible kidney injury”.

Author Response: Dear Reviewer, thank you for your remarks. It has been corrected in the revised manuscript.

Comment #9: Line 88: replace “abnormal increased levels of blood urea and creatinine” to “abnormally elevated levels of blood urea and creatinine”.

Author Response: Dear Reviewer, thank you for your suggestion. It has been replaced.

Comment #10: Line 83: Insert the statement from Line 90-92 “In the ……[20]” in line 83 after the reference “[16]”.

Author Response: Dear Reviewer, thank you for your suggestion. It has been considered in the revised manuscript.

Comment #11: Lines 101-105: this objective is not the same as in the abstract section – make it uniform/the same.

Author Response: Dear Reviewer, thank you for your remark. The objective has been revised to make it uniform with the one stated in the abstract.

Methods

Comment #12: Lines 111-113: what is the association between Cd levels/exposures with seasons? Did you show that in this work? I though population size of Buea could be vital mentioning here, so readers understand the scope of the study and potential damage due to Cd from smoking – or better still insert the data on population size

Author Response: Thank you for this important observation. We agree that seasonal variation could influence environmental cadmium exposure, particularly through factors such as rainfall, humidity, and agricultural burning, which may affect ambient air quality and tobacco leaf cadmium content. However, in this study, we did not assess the association between cadmium exposure levels and seasons. Our investigation was a cross-sectional study conducted over a single time frame (May to October 2024), which corresponds to the long rainy season in Buea. Therefore, we were unable to account for seasonal variations throughout the year. We have now clarified this limitation in the revised manuscript.

Regarding the population size of Buea, we agree that providing this information helps readers better understand the scope and generalizability of the study. Buea is the capital of the Southwest Region of Cameroon and had an estimated population of approximately 250,000–300,000 inhabitants as of recent estimates, with a high proportion of young adults due to the presence of the University of Buea. We have now added the population data in the Study area section to contextualize the study setting and the potential public health impact of cadmium exposure from cigarette smoking in this community.

Comment #13: Lines 115-117: the statement “The area was therefore chosen to raise awareness among the young and old alike that smoking can potentially damage vital organs like the kidneys and the liver.” appears as if you knew the results of this work and as if the aim of your work was to raise awareness! I suggest you rephrase.

Author Response: Dear Reviewer, thank you for your comment. The sentence has been rephrased in the revised manuscript as follows: …The area was therefore selected due to its large young adult population, enabling us to evaluate smoking-induced liver and kidney damage, with the goal of using the findings to guide future public health awareness efforts…

Comment #14: Lines 118-122: some vital information/components are missing on the study design, for example, the variables (independent/dependent), sampling methods, etc. kindly consider re-writing this section.

Author Response: Dear Reviewer, thank you for your comments. We have rewritten the section and the missing information/components have been included in the revised manuscript. Please, refer to Page 5 and 6.

Comment #15: Lines 124-125: if that was the primary aim, what about the effects on hepatocellular/kidney functions etc

Author Response: Thank you for this important observation. You are correct that the original wording of the primary aim was incomplete, as it only mentioned estimating cadmium exposure levels without including the assessment of effects on liver and kidney functions, which is a major component of this study. We have now revised the sentence to clearly state that the study had two primary aims: (1) to estimate cadmium exposure from cigarette smoking, and (2) to assess the association between smoking and abnormal levels of liver and kidney function biomarkers (including hepatocellular injury markers such as ALT, and renal function markers such as urea, creatinine, albuminuria, and glucosuria).

Comment #16: Line 129: is this “Cameroon [23] (Njoumeni et al., 2020)” or “Cameroon [23]” or “Cameroon [23, 24]” reference 24 being Njoumeni et al., 2020?

Author Response: Dear Reviewer, thank you for your remark. The correct citation is “Cameroon [23]”. “(Njoumeni et al., 2020)” has been deleted.

Comment #17: Line 131: N of 78 (95) is too small for such studies! Well, lets deal with it.

Author Response: Dear Reviewer, thank you for this observation. We acknowledge that a sample size of 95 active smokers (derived from 78 plus 20%) may appear relatively small for epidemiological studies. However, we would like to provide the following justifications:

Prevalence-based calculation: The sample size was calculated using Cochran's formula based on the reported smoking prevalence in Cameroon (5.3%). This is a standard approach for cross-sectional studies when the population size is large and no prior data on the specific outcome are available.

Feasibility constraints in resource-limited settings: This study was conducted in Buea, Cameroon, where resources for large-scale biomonitoring studies are limited. The recruitment of 102 active smokers and 37 non-smokers (total 139 participants) was practically achievable within the study period and budget, while still allowing for meaningful statistical comparisons.

Limitation acknowledged: We agree that a larger sample size would enhance generalizability and allow for more robust subgroup analyses. We have now explicitly acknowledged this as a limitation in the revised manuscript (Discussion section). We have included the following sentence to the limitations paragraph: “Furthermore, although the sample size was calculated based on the local smoking prevalence and was sufficient to detect significant associations, it remains relatively modest. A larger, multi-center study would be necessary to confirm these findings and improve their generalizability to the broader Cameroonian population”.

Comment #18: Lines 133-137: Inclusion and exclusion criteria not well stated eg., did you include smokers with known illnesses? Line 140: Are there smoking areas in Buea??? Line 140: replace “a” with “each”

Author Response: Dear Reviewer, thank you for your comments. Sub-headings have been included in the revised manuscript to specifically include the “inclusion and exclusion criteria”. There are no smoking areas in Buea and the statement has been deleted. Line 140: replace “a” with “each”, corrected.

Comment #19: Lines 141-142: Not only is the statement too long, it has repetitions e.g. gave their consent by signing the informed consent … therefore, kindly shorten the statement and rephrase the second part of that statement from “..were clearly explained; and only participants who gave their consent by signing the informed consent were admitted to the study.” to read as “..were clearly explained. Participation in the study was limited to individuals who signed the informed consent form.”

Author Response: Dear Reviewer, thank you for your remark. The statement has been corrected as suggested.

Comment #20: Lines 138-149: What were the inclusion and exclusion criteria? This is missing from the manuscript. This information was attempted on lines 118-122.

Author Response: Dear Reviewer, thank you for your remark. The inclusion and exclusion criteria have been explicitly added. Please, refer to Page 6 of the revised manuscript.

Comment #21: Line 154: How was “pre-testing” done? I suggest you provide a statement on that. For example – “During the pre-testing phase, the questionnaire was randomly administered to five market women and ten primary school pupils in classes five and six. This process aimed to ensure that the language used in the questionnaire was simple, clear, and unambiguous, and that it could be easily understood by all participants. As more than 80% positive responses were obtained, the questionnaire was considered valid. The results of the pre-testing phase were not included in the findings of this study.”

Author Response: Dear Reviewer, thank you for your comment. We have now added a description of pre-testing phase according to your suggestions. Thank you very much for that useful suggestion.

Comment #22: Lines 151-163: The heading on line 151 does not adequately reflect the content in lines 152-163. Lines 158-163 deserve a separate subheading “Sampling of biological specimens (blood and urine)”. Meanwhile, the square brackets in Line 158 needs to be adjusted.

Author Response: Thank you for this careful

Attachment Submitted filename: Response to Reviewers.docx

# Peer review: Decision Letter 1

**DOI:** 10.1371/journal.pone.0333775.r003

**Authors:** Iman Al-Saleh

8 May 2026

PONE-D-25-50711R1

Cadmium Exposure and Hepato-renal Injury in Response to Cigarette Smoking in Apparently Healthy Active Smokers in Buea-Cameroon

PLOS One

Dear Dr. Kouam,

Thank you for submitting your manuscript to PLOS ONE. After careful consideration, we feel that it has merit but does not fully meet PLOS ONE’s publication criteria as it currently stands. Therefore, we invite you to submit a revised version of the manuscript that addresses the points raised during the review process.

Thank you for revising the manuscript and carefully addressing most of the reviewers’ comments, including the minor revisions suggested by Reviewer #1. The manuscript has improved substantially.

However, before the manuscript can be considered for acceptance, please carefully address the remaining concerns raised by Reviewer #2, particularly regarding the interpretation of cadmium-specific effects in the context of cigarette smoke as a complex mixture of toxicants. The title and relevant sections of the manuscript should be revised accordingly to avoid overstating cadmium as the sole causative factor.

Please submit your revised manuscript by Jun 22 2026 11:59PM. If you will need more time than this to complete your revisions, please reply to this message or contact the journal office at plosone@plos.org. When you're ready to submit your revision, log on to https://www.editorialmanager.com/pone/ and select the 'Submissions Needing Revision' folder to locate your manuscript file.

Please include the following items when submitting your revised manuscript:

- A letter that responds to each point raised by the academic editor and reviewer(s). You should upload this letter as a separate file labeled 'Response to Reviewers'.
- A marked-up copy of your manuscript that highlights changes made to the original version. You should upload this as a separate file labeled 'Revised Manuscript with Track Changes'.
- An unmarked version of your revised paper without tracked changes. You should upload this as a separate file labeled 'Manuscript'.

If you would like to make changes to your financial disclosure, please include your updated statement in your cover letter. Guidelines for resubmitting your figure files are available below the reviewer comments at the end of this letter.

If applicable, we recommend that you deposit your laboratory protocols in protocols.io to enhance the reproducibility of your results. Protocols.io assigns your protocol its own identifier (DOI) so that it can be cited independently in the future. For instructions see: https://journals.plos.org/plosone/s/submission-guidelines#loc-laboratory-protocols. Additionally, PLOS ONE offers an option for publishing peer-reviewed Lab Protocol articles, which describe protocols hosted on protocols.io. Read more information on sharing protocols at https://plos.org/protocols?utm_medium=editorial-email&utm_source=authorletters&utm_campaign=protocols.

As the corresponding author, your ORCID iD is verified in the submission system and will appear in the published article. PLOS supports the use of ORCID, and we encourage all coauthors to register for an ORCID iD and use it as well. Please encourage your coauthors to verify their ORCID iD within the submission system before final acceptance, as unverified ORCID iDs will not appear in the published article. *Only* the individual author can complete the verification step; PLOS staff *cannot* verify ORCID iDs on behalf of authors.

We look forward to receiving your revised manuscript.

Kind regards,

Iman Al-Saleh

Academic Editor

PLOS One

Journal Requirements:

If the reviewer comments include a recommendation to cite specific previously published works, please review and evaluate these publications to determine whether they are relevant and should be cited. There is no requirement to cite these works unless the editor has indicated otherwise.

Please review your reference list to ensure that it is complete and correct. If you have cited papers that have been retracted, please include the rationale for doing so in the manuscript text, or remove these references and replace them with relevant current references. Any changes to the reference list should be mentioned in the rebuttal letter that accompanies your revised manuscript. If you need to cite a retracted article, indicate the article’s retracted status in the References list and also include a citation and full reference for the retraction notice.

[Note: HTML markup is below. Please do not edit.]

Reviewers' comments:

Reviewer's Responses to Questions

**Comments to the Author**

1. If the authors have adequately addressed your comments raised in a previous round of review and you feel that this manuscript is now acceptable for publication, you may indicate that here to bypass the “Comments to the Author” section, enter your conflict of interest statement in the “Confidential to Editor” section, and submit your "Accept" recommendation.

Reviewer #2: (No Response)

**********

2. Is the manuscript technically sound, and do the data support the conclusions?

The manuscript must describe a technically sound piece of scientific research with data that supports the conclusions. Experiments must have been conducted rigorously, with appropriate controls, replication, and sample sizes. The conclusions must be drawn appropriately based on the data presented.

Reviewer #2: Partly

**********

3. Has the statistical analysis been performed appropriately and rigorously?

Reviewer #2: Yes

**********

4. Have the authors made all data underlying the findings in their manuscript fully available?

The PLOS Data policy requires authors to make all data underlying the findings described in their manuscript fully available without restriction, with rare exception (please refer to the Data Availability Statement in the manuscript PDF file). The data should be provided as part of the manuscript or its supporting information, or deposited to a public repository. For example, in addition to summary statistics, the data points behind means, medians and variance measures should be available. If there are restrictions on publicly sharing data—e.g. participant privacy or use of data from a third party—those must be specified.

Reviewer #2: Yes

**********

5. Is the manuscript presented in an intelligible fashion and written in standard English?

PLOS ONE does not copyedit accepted manuscripts, so the language in submitted articles must be clear, correct, and unambiguous. Any typographical or grammatical errors should be corrected at revision, so please note any specific errors here.

Reviewer #2: Yes

**********

6. **Review Comments to the Author**

Please use the space provided to explain your answers to the questions above. You may also include additional comments for the author, including concerns about dual publication, research ethics, or publication ethics. (Please upload your review as an attachment if it exceeds 20,000 characters)

Reviewer #2: In context of the presence of co-toxicants in addition to Cadmium (Cd) in cigarette smoke as PAHs

(Polycyclic Aromatic Hydrocarbons) e.g. B(a)P: Benzo (a) pyrene, B(a)A: Benzo (a) anthracene and

TSNAs (Tobacco Specific Nitrosamines) e.g. NNN: Nitrosonornicotine, NNK:4(methylnitrosamine)-1-(3-pyridyl)-1-butanone etc and Volatile components as CO, hydrocyanide, methane, ethylene, phenol, 1,3-butadiene etc. The cadmium exposure stands as one of the contributing factor for the observed hepato-renal injury in apparently healthy individuals.

1. It is recommended that the “Title” should be changed as:

“Cadmium exposure as a contributing factor causing hepato-renal Injury in apparently healthy active cigarette smokers in Buea-Cameroon.”

2. Authors must acknowledge and explicitly write and incorporate it at various places (e.g. in Abstract, Introduction, Methods and Discussion) in addition to cadmium, presence of PAHs and aromatic amines etc. (as mentioned above) as co-toxicants and confounders. It should be stated that combined exposure may likely to potentiates/contribute organ dysfunction. However, in a resource-limited setting (Cameroon), this study acknowledges a significant limitation to evaluate the effect of other co-toxicants on liver and kidney. The present revolves around evaluation of cadmium exposure of individuals while active cigarette smoking and its effect on hepatic and renal functions.

3. In the conclusion, it should be added that to distinguish cadmium-specific effects from effects caused by PAHs and aromatic amines would require additional biomonitoring (e.g., blood and urinary cadmium levels), mechanistic biomarkers (e.g., metallothionein, β2microglobulin), and DNA adduct analysis [4,5]. Additional bioassays incorporating these measurements are needed to identify the specific contribution of cadmium to smoking-induced liver and kidney injury. Exposure to cadmium may cause carcinogenesis/mutagenesis via CYP 450 activation and may not directly form stable DNA adduct but may induce oxidative stress and interfere with DNA repair mechanisms.

**********

7. PLOS authors have the option to publish the peer review history of their article (what does this mean?). If published, this will include your full peer review and any attached files.

If you choose “no”, your identity will remain anonymous but your review may still be made public.

**Do you want your identity to be public for this peer review?** For information about this choice, including consent withdrawal, please see our Privacy Policy.

Reviewer #2: **Yes:** Lakshmi Bala

**********

[NOTE: If reviewer comments were submitted as an attachment file, they will be attached to this email and accessible via the submission site. Please log into your account, locate the manuscript record, and check for the action link "View Attachments". If this link does not appear, there are no attachment files.]

To ensure your figures meet our technical requirements, please review our figure guidelines: https://journals.plos.org/plosone/s/figures

You may also use PLOS’s free figure tool, NAAS, to help you prepare publication quality figures: https://journals.plos.org/plosone/s/figures#loc-tools-for-figure-preparation.

NAAS will assess whether your figures meet our technical requirements by comparing each figure against our figure specifications.

Attachment Submitted filename: Lakshmi Bala_comments.docx

# Author comment: Author response to Decision Letter 2

**DOI:** 10.1371/journal.pone.0333775.r004

21 May 2026

RESPONSE TO THE REVIEWER’S COMMENTS

Note: All NEW changes or suggestions are written in Green in the revised manuscript

Dear Editor-in-Chief of the journal: PLOS ONE

Thank you for your letter dated May 08th, 2026, and the opportunity given to us to revise and resubmit the manuscript entitled “Cadmium Exposure and Hepato-renal Injury in Response to Cigarette Smoking in Apparently Healthy Active Smokers in Buea-Cameroon. PONE-D-25-50711”. We would also like to take this opportunity to thank the editorial team and reviewers for their helpful comments, which greatly contributed to improving the current version of this manuscript. The manuscript has been updated in accordance with the reviewers' recommendations. Most of their inquiries have been answered, and some clarifications have been provided. Throughout this revised version of the manuscript, new modifications are written in green.

Reviewer’s comment

Reviewer #2: In context of the presence of co-toxicants in addition to Cadmium (Cd) in cigarette smoke as PAHs (Polycyclic Aromatic Hydrocarbons) e.g. B(a)P: Benzo (a) pyrene, B(a)A: Benzo (a) anthracene and TSNAs (Tobacco Specific Nitrosamines) e.g. NNN: Nitrosonornicotine, NNK:4(methylnitrosamine)-1-(3-pyridyl)-1-butanone etc and Volatile components as CO, hydrocyanide, methane, ethylene, phenol, 1,3-butadiene etc. The cadmium exposure stands as one of the contributing factor for the observed hepato-renal injury in apparently healthy individuals.

Comment #1: It is recommended that the “Title” should be changed as:

“Cadmium exposure as a contributing factor causing hepato-renal Injury in apparently healthy active cigarette smokers in Buea-Cameroon.”

Author Response: We thank the reviewer for this important suggestion. We agree that the revised title more accurately reflects that cadmium is one of several contributing factors to hepato-renal injury, rather than the sole cause. We have therefore changed the title as recommended.

Comment #2: Authors must acknowledge and explicitly write and incorporate it at various places (e.g. in Abstract, Introduction, Methods and Discussion) in addition to cadmium, presence of PAHs and aromatic amines etc. (as mentioned above) as co-toxicants and confounders. It should be stated that combined exposure may likely to potentiate/contribute organ dysfunction. However, in a resource-limited setting (Cameroon), this study acknowledges a significant limitation to evaluate the effect of other co-toxicants on liver and kidney. The present revolves around evaluation of cadmium exposure of individuals while active cigarette smoking and its effect on hepatic and renal functions.

Author Response: We have carefully incorporated this acknowledgment at multiple sections of the manuscript as requested. Specific additions have been made to the Abstract, Introduction, Methods, and Discussion sections. Throughout the revised manuscript, modifications are written in green.

Comment #3: In the conclusion, it should be added that to distinguish cadmium-specific effects from effects caused by PAHs and aromatic amines would require additional biomonitoring (e.g., blood and urinary cadmium levels), mechanistic biomarkers (e.g., metallothionein, β2microglobulin), and DNA adduct analysis [4,5]. Additional bioassays incorporating these measurements are needed to identify the specific contribution of cadmium to smoking-induced liver and kidney injury. Exposure to cadmium may cause carcinogenesis/mutagenesis via CYP 450 activation and may not directly form stable DNA adduct but may induce oxidative stress and interfere with DNA repair mechanisms.

Author Response: Dear Reviewer, thank you for your suggestions. We have added the recommended text to the Conclusion section.

The authors are grateful to the reviewers for their valuable contributions which significantly improved the quality of this work. We very much hope the revised manuscript is accepted for publication in your Journal. Thank you for your consideration.

Sincerely yours,

Corresponding author

Kouam Fondjo Arnaud

Attachment Submitted filename: Response_to_Reviewers_auresp_2.docx

# Editor report: Decision Letter 2

**DOI:** 10.1371/journal.pone.0333775.r005

**Authors:** Iman Al-Saleh

25 May 2026

Cadmium Exposure as a Contributing Factor Causing Hepato-Renal Injury in Apparently Healthy Active Cigarette Smokers in Buea-Cameroon

PONE-D-25-50711R2

Dear Dr. Kouam,

The reviewers’ and editorial comments have been adequately addressed, and I am pleased to inform you that the manuscript is acceptable for publication pending final production checks. During proof correction, please revise the title slightly by deleting the word “causing” to avoid implying direct causality. The title should read: **Cadmium Exposure as a Contributing Factor to Hepato-Renal Injury in Apparently Healthy Active Cigarette Smokers in Buea-Cameroon**

Within one week, you’ll receive an e-mail detailing the required amendments. When these have been addressed, you’ll receive a formal acceptance letter and your manuscript will be scheduled for publication.

An invoice will be generated when your article is formally accepted. Please note, if your institution has a publishing partnership with PLOS and your article meets the relevant criteria, all or part of your publication costs will be covered. Please make sure your user information is up-to-date by logging into Editorial Manager at Editorial Manager® and clicking the ‘Update My Information' link at the top of the page. For questions related to billing, please contact billing support.

If your institution or institutions have a press office, please notify them about your upcoming paper to help maximize its impact. If they’ll be preparing press materials, please inform our press team as soon as possible -- no later than 48 hours after receiving the formal acceptance. Your manuscript will remain under strict press embargo until 2 pm Eastern Time on the date of publication. For more information, please contact onepress@plos.org.

Kind regards,

Iman Al-Saleh

Academic Editor

PLOS One

Additional Editor Comments (optional):

Reviewers' comments:

# Editor report: Acceptance letter

**DOI:** 10.1371/journal.pone.0333775.r006

**Authors:** Iman Al-Saleh

PONE-D-25-50711R2

PLOS One

Dear Dr. Kouam,

I'm pleased to inform you that your manuscript has been deemed suitable for publication in PLOS One. Congratulations! Your manuscript is now being handed over to our production team.

At this stage, our production department will prepare your paper for publication. This includes ensuring the following:

* All references, tables, and figures are properly cited

* All relevant supporting information is included in the manuscript submission,

* There are no issues that prevent the paper from being properly typeset

You will receive further instructions from the production team, including instructions on how to review your proof when it is ready. Please keep in mind that we are working through a large volume of accepted articles, so please give us a few days to review your paper and let you know the next and final steps.

Lastly, if your institution or institutions have a press office, please let them know about your upcoming paper now to help maximize its impact. If they'll be preparing press materials, please inform our press team within the next 48 hours. Your manuscript will remain under strict press embargo until 2 pm Eastern Time on the date of publication. For more information, please contact onepress@plos.org.

You will receive an invoice from PLOS for your publication fee after your manuscript has reached the completed accept phase. If you receive an email requesting payment before acceptance or for any other service, this may be a phishing scheme. Learn how to identify phishing emails and protect your accounts at https://explore.plos.org/phishing.

If we can help with anything else, please email us at customercare@plos.org.

Thank you for submitting your work to PLOS ONE and supporting open access.

Kind regards,

PLOS ONE Editorial Office Staff

on behalf of

Dr. Iman Al-Saleh

Academic Editor

PLOS One
